# Self-adaptative Early Warning Scoring System for Smart Hospital

**DOI:** 10.1007/978-3-030-51517-1_2

**Published:** 2020-05-31

**Authors:** Imen Ben Ida, Moez Balti, Sondès Chabaane, Abderrazak Jemai

**Affiliations:** 8 Digital Research Centre of Sfax, Sfax, Tunisia; 9 Institut Mines-Télécom, CNRS, Paris, France; 10 Université de Sherbrooke, Sherbrooke, QC Canada; 11 Digital Research Centre of Sfax, Sfax, Tunisia; 12 University of Sfax, Sfax, Tunisia; 13 Electronic Systems and Communications Networks Laboratory (SERCOM), Polytechnic School of Tunisia, Carthage University, Tunis, Tunisia; 14 IsetCom, Carthage University, Tunis, Tunisia; 15 CNRS UMR 8201 - LAMIH - Laboratory of Automatic Mechanics and Industrial and Human Informatics, Hauts-de-France Polytechnic University, 59313 Valenciennes, France; 16 INSAT, Carthage University, Tunis, Tunisia

**Keywords:** Computing for healthcare, Early warning scoring system, Internet of Things, Smart hospital

## Abstract

With the advent of the Internet of Things (IoT), various interconnected objects can be used to improve the collection and the process of vital signs with partially or fully automatized methods in smart hospital environment. The vital signs data are used to evaluate patient health status using heuristic approaches, such as the early warning scoring (EWS) approach. Several applications have been proposed based on the early warning scores approach to improve the recognition of patients at risk of deterioration. However, there is a lack of efficient tools that enable a personalized monitoring depending on the patient situations. This paper explores the publish-subscribe pattern to provide a self-adaptative early warning score system in smart hospital context. We propose an adaptative configuration of the vital sings monitoring process depending on the patient health status variation and the medical staff decisions.

## Introduction

The smart hospital (SH) is adding the intelligence to traditional hospital system to improve the quality of healthcare services. It is based on the effective use of technology and it covers all the resources and the locations with the patient information. A principal functionality in a smart hospital is the automated and continuous control of the patients during the hospitalization by measuring their vital signs. These observations are important for preventing health deterioration, reducing costs and hospitalization time, and potentially minimizing morbidity and mortality [1]. Several medical approaches are used to evaluate the collected vital sings data. A prevalent example is the Early warning scoring (EWS) approach which has been in use for several years as a tool to predict the risk level of patients [2]. It was proposed for the first time as a paper-based method that need periodical checkups to assign a score based on patient’s vital signs (i.e., heart rate, respiration rate, body temperature, blood pressure). The score of each medical sign depends on the non-respect of a predefined normal interval. The summation of all scores reflects the global patients risk level [2].

By exploring the Internet of Things technologies, the vital signs control solutions are automated based on various medical devices and sensors. Smart medical devices constitute the core part of the smart hospital environment. Their main purpose is to gather vital signs data or other patient physiological conditions. These automated systems reduce the errors of the manual Early Warning Score systems [3] and facilitate the nurses’ functions such as constantly gathering and storing the vital signs records.

The emergence of the Internet of Things, the electronic records and the computerized transaction systems have improved the efficiency and effectiveness of EWS systems.

In spite of such advantages using IoT, there are currently two important challenges of early warning scores systems and they need to be considered. The first issue is how to ensure a personalized monitoring of patients’ vital signs depending on their situations and the medical experts’ requirements especially in case of controlling an important number of patients.

The second issue is the need of timely response of medical staff in case of problem detection.

Our work is motivated by the challenges described above and its main objective is a self-adaptative Early Warning Scoring system that supports the change of the patient control frequency depending on his situation.

This paper is organized as follows: In Sect. 2 we describe the vital signs evaluation with the early warning score systems and we present some related works. Our proposed solution is detailed in Sect. 3. The Implementation and the evaluation are presented and discussed in Sect. 4. The Sect. 5 presents the concluding remarks and future work.

## Background and Related Works

Early warning systems, also known as ‘track-and-trigger’ (T&T) systems, consist of evaluating vital sings using scores to recognize patients at risk of deterioration. Since 85% of severe adverse events (SAE) are preceded by abnormal vital signs, the vital signs monitoring based on EWS approach have evolved as a means of alerting health professionals to patient clinical decline [4].

In smart hospitals, particularly in intensive care units, the Early Warning Score (EWS) is a prevalent tool, by which patient’s vital signs are periodically recorded and the emergency level is interpreted [3]. To this end, a score (0 for a perfect condition and 3 for the worst condition) is allocated to each vital sign according to its value and the predefined limits. The summation of the obtained scores indicates the degree of health deterioration of the patient (the higher the EWS, the worse the patient’s health condition).

### National Early Warning Scoring (NEWS)2 Approach

**Fig. 1.**
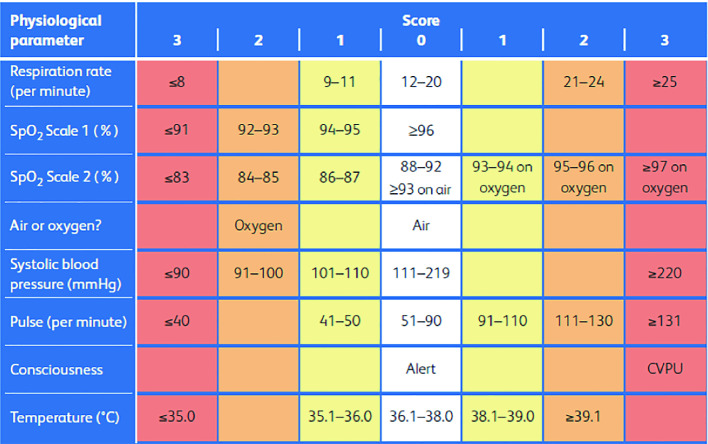
The NEWS2 scores chart.

NEWS2 chart illustrated in Fig. 1 is a revised version of NEWS chart. The NEWS was created to standardize the process of recording, scoring and responding to changes in routinely measured physiological parameters in acutely ill patients. It was developed to improve the detection of clinical deterioration in patients with acute illness. In [5] NEWS was evaluated against a range of outcomes that are of major importance to patients and staff. It demonstrates a good ability to discriminate patients at risk of the combined outcome of cardiac arrest, unanticipated ICU admission or death within 24 h, which provides ample opportunity for an appropriate clinical intervention to change patient outcome [6].

NEWS2 could be made safer for patients with hypercapnic respiratory failure by having two scoring systems for (saturation pulse oxygen) SpO2:

- The existing SpO2 scoring system (Scale 1) that would apply to the majority of patients.
- A dedicated SpO2 scoring system for patients with hypercapnia respiratory failure (Scale 2). This illness means that the patient doesn’t have enough oxygen in his blood and his desired oxygen saturations are set at a lower level (88–92%). The NEWS scoring system is adjusted accordingly.

### Early Warning Score Systems Requirements

Early warning score systems are used to improve the process of recording, scoring and responding to changes in measured vital signs of patients. The following paragraphs present the principal requirements of EWS systems to ensure an efficient detection and response to clinical deterioration:

- Personalization

The patient vital sings data are the key of successful medical decisions. Each patient requires a personalized control depending on his situation [7]. For example a patient with a disease that causes hyperthermia, the temperature is a critical data which must be visualized with more precision; every second. While other non-critical parameters can be calculated only every hour for example.

- Medical staff engagement

The early warning scores systems are highly user-dependent and depend on the appropriate response and actions of the Medical Emergency Teams [4].

- Need for expert’s decision

The warning scores cannot replace specialist’s decision and the importance on knowing individual patients, they cannot also replace the background to the observations that are recorded [2].

NEWS correlated poorly with the patient’s clinical status within the first 24 h post-operatively [2].

### Related Works

In [8], authors present a solution which takes benefits from the concept of Edge computing and fog computing in the context of IoT based Early Warning Scores systems. The solution provides high level services in a Geo-distributed fashion at the edge of the network. The proof of concept is demonstrated with smart e-Health Gate-way called UT-GATE implemented for an IoT-based remote health monitoring system. The demonstration includes the data flow processes from data acquisition at sensor nodes to the cloud and the end-users. However, the data has a static interval of acquisition which is defined at the development stage. As a result, the solution does not give the possibility of personalization. The smart e-Health Gateway proposed in [9] provides local storage to perform real-time local data processing and mining. When a patient’s vital signal is processed, reliable IoT systems are provided to facilitate fault-tolerant healthcare services. Zhang et al. proposes in [10] a patient-centric cyber-physical system named Health-CPS aiming to ensure convenient Healthcare service. The Health-CPS depends on Cloud computing and data analytics to handle the big data related issues of different healthcare applications. The system is composed of several layers such as data collection layer, data management layer and data-oriented service layer. The system collects data in a unified standard. It supports distributed storage and parallel processing.

The authors present in [11] a low-cost health monitoring system that provides continuous remote monitoring of the ECG and automatic analysis and notification. The system consists of energy-efficient sensor nodes and a fog layer that take full advantage of IoT. The sensor nodes collect and wirelessly transmit ECG, respiration rate, and body temperature to a smart gateway which can be accessed by appropriate care-givers. In addition, the system can represent the collected data in useful ways, perform automatic decision making and provide many advanced services such as real-time notifications for immediate attention.

All the previous proposed solutions require different technology skills to modify or to scale out an existing system in a hospital context. The devices list is predefined at the conception level with fixed parameters of configuration. Added to that, the frequency of collecting and saving data are defined by the developers at the level of implementation without the possibility of modification after the deployment in a real case. As a result of this static configuration, a device collect data with the same parameters defined from the first step of development and it cannot be reused for personalized cases.

However, patient data are different from other collected data in IoT environments as it depends on the no stable situation of the patient health. For example, the frequency of the collected respiration data with a sensor depends on the patient illness. In some cases, an interval of 1 h is sufficient but in other cases 1 min is needed. If the storage operation of data is unique, an important unnecessary information will take place in the memory and demand useless process. In this paper, we propose a health-care system that makes it easy to personalize the vital signs monitoring of a particular patient depending on his health condition.

## The Proposed Solution

Our proposed solution supports a patient-driven process by giving the possibility to adjust the parameters of vital signs monitoring to a specific health condition or treatment. The following paragraphs describe the solution architecture and the supported communication model.

### Publish-Subscribe Communication Model

Our solution uses the publish/subscribe pattern for the data exchange between the different architecture layers. Publish-subscribe messaging systems support data-centric communication and have been widely used in IoT systems. With the publish-subscribe pattern, the exchange of messages between clients is ensured using a broker that manages topics and sub-topics. A publisher on a given topic can send messages to other clients acting as subscribers to the topic without the need to know about the existence of the receiving clients [5].

To organize the topics and sub-topics in both gateway and server brokers, we define four categories of data:

- Sensed data: The collection of time-series data sensed by the medical devices.
- EWS score data: The corresponding score of each sensed data depending on the NEWS2 specification illustrated in Fig. 1.
- Configuration data: The definition of the gateway parameters by the medical staff. For example, the frequency of saving the collected data.

### Gateway Layer Components

**Fig. 2.**
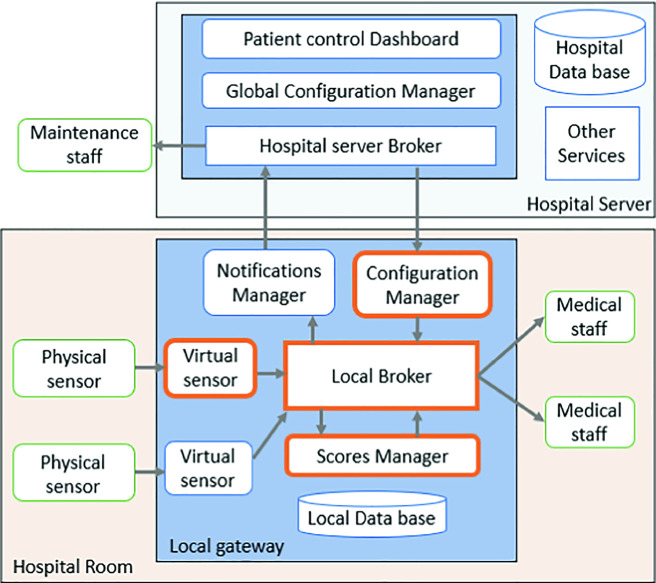
The solution architecture.

We propose the use of a gateway as an edge layer to put the computing at the proximity of data sources. In each hospital room, a smart gateway is used to collect the vital signs of the patients. The main components of the proposed gateway illustrated in Fig. 2 are:

**a) Local broker**

The Broker server acts as an intermediary for messages sent between the publishers and the subscribers for a specific topic. It routes the messages based on topic rather than the IP address. When a message is sent by the publish client to the broker server, all the subscribe clients interested on the related topic of the message will receive the publication.

Medical devices are considered as data publishers. A topic is the device name and for each device, a subscriber is created to listen to the published data and save it in a local database with the required parameters. The main component of the gateway is an embedded configuration service that provides medical staff with the ability to log into and configure the data process behavior depending on patients’ situations. Medical staff can configure publishers and subscribers by providing information such as the ID of the devices, the references of the patients and the corresponding dates of hospitalization.

**b) The virtual sensor**

It is a software component that has three main roles. First, it saves the received data as significant information in the local database. Second, it calculates the score of the data depending on its type and using the predefined intervals of the NEWS2 approach. In case of problem detection with the calculated score or data interruption, the virtual sensor publishes a notification to the local broker. Added to that, each virtual sensor is subscribed to a configuration topic in order to receive the requested interval of saving data in the local database. To ensure the privacy of the patient information, the virtual sensor does not store the patients’ details such as their names or their ages. It stores only the references of patients associated with the devices’ numbers and the corresponding range time of data collection.

**c) Scores manager**

The scores manager is a software component that receive calculated scores from all the virtual sensors. It calculates the global score of each patient and evaluates its risk level. The calculated scores are saved in the local database and they are sent regularly to the hospital server for long term storage. In case of high score detection, the scores manager keep publishing significant notifications to the local broker until it receives a validation of the medical staff intervention.

**d) Configuration manager**

The configuration manager is in an intermediate position between the hospital server and the virtual sensors. It initializes each new virtual sensor with the corresponding configuration. The configuration manager of each gateway is considered as a subscriber to the corresponding configuration topic defined at the Hospital server.

### Methods

The main objective of our solution is to provide intelligent methods of patient control. Two types of configuration are supported by the configuration manager:

- Devices configuration

The medical staff can submit a configuration request using a web interface, the server broker publish the received request as a configuration message to the concerned gateway. If the request is about adding a new device, a new virtual sensor is created after validation. In case that the medical staff choose to modify some parameters of an existing device, the corresponding virtual sensor checks the different inserted parameters: device identifier, patient reference and the time range of saving data and save the new configuration. The separation of the virtual sensors as independent subscribers ensures that the modification or the elimination of a device does not affect the whole system.

- Data management configuration

Added to the devices configuration possibility, our system integrates an automation algorithm for the calibration of the data storage and the score calculation frequency.

For each score level, the maximum value is defined as score level limit. The configuration manager reads frequently the calculated score of each vital sign from the local database and compare it with the corresponding predefined limits.

If it captures successive abnormal values, the frequency of the collection of this data is automatically increased to be able to monitor the data concerned with more precision. The new frequency value is saved in the local database and published as a message to the corresponding virtual sensor.

When the measurements become more stable and less than the predefined limits, the administrator can decide to change this frequency.

**Fig. 3.**
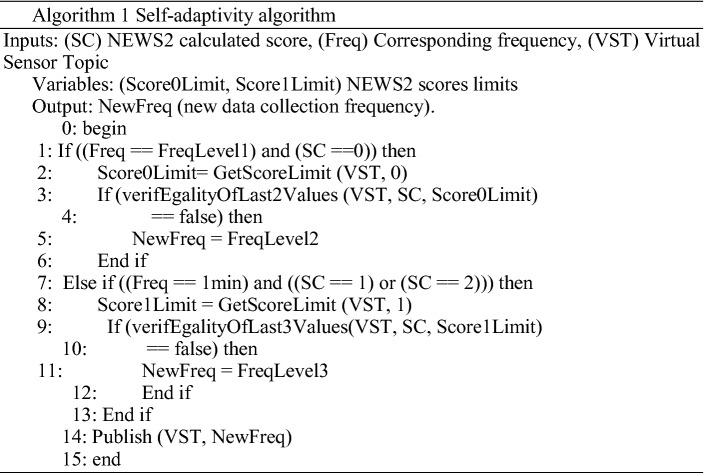
Self-adaptative configuration algorithm.

The values of required frequencies are defined depending on the medical experts’ requirements (Fig. 3).

The self-adaptivity process is illustrated in the following algorithm:

## Implementation and Evaluation

### Materials

The Gateway is a Raspberry Pi 3 which is a small size board with 1 GB of Ram and 1.2 GHz processor [12]. As an Operating System for the gateway, we use Raspbian the pre-compiled Debian OS which is especially optimized for the Raspberry Pi.

We install the InfluxDB database which is an open-source Time Series Database written in Go. At its core is a custom-built storage engine called the Time-Structured Merge (TSM) Tree, which is optimized for time-series data. InfluxDB supports SQL-like query languages named InfluxQL and has the advantage of easy scale-out. It provides support for mathematical and statistical functions across time ranges, also it is developed for custom monitoring, metrics collection and real-time analytics [13].

We use the mosquito implementation as a messages’ broker and Node JS clients as subscribers [14].

The messages exchange is ensured by MQ Telemetry Transport (MQTT) protocol [15]. MQTT protocol is a lightweight application layer protocol designed for resource-constrained devices. It runs over TCP/IP, or over other network protocols that provide ordered, lossless and bidirectional connections. It uses the publish/subscribe messaging system combined with the concept of topics to provide one-to-many message distribution. The headers of MQTT messages are small and the connection set up does not require a synchronous handshake which could support a range of 10 to 100 messages per second.

MQTT applies topic-based filtering of messages with a topic being part of each published message. The broker uses the topics to determine whether a subscribing client should receive the message or not. Clients can subscribe to as many topics as they are interested in.

### Evaluation and Results

The evaluation of the presented solution was done from a resource use point of view to analyze whether the self-adaptative algorithm would result in a better performance parameter. The parameters that were taken into account were memory use for stored data and the CPU use of the gateway.

To prove the benefits of the customized use of the gateway, we consider a scenario of controlling the temperature data of one patient with two different scenarios. The first is called fixed case, it is the standard case in which the data are collected with a unique interval of time. In the second case, the interval of data collection changes depending on the patient’s score calculation.

**Fig. 4.**
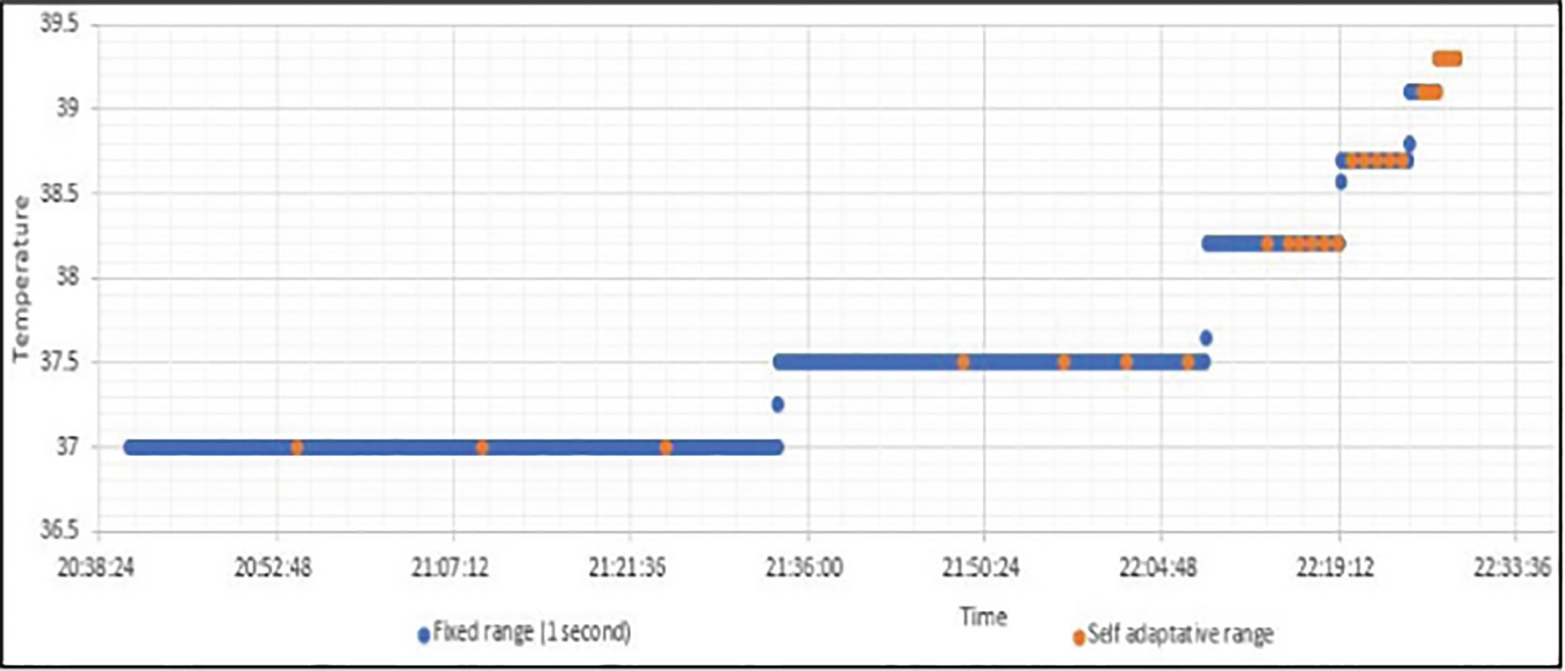
Self-adaptative configuration example.

In Fig. 4, we illustrate a result of the self-adaptivity configuration. We support 3 levels of data storage frequency depending on the corresponding score. The green signal presents the temperature measurements of a patient. The frequency of saving the sensed data changes when successful augmentation of temperature value is detected. The second signal which does not respect the self-adaptative algorithm contains unnecessary information for the first seven hour and before the increase of patient’s temperature.

We simulate 5 devices that send temperature values ​​using the MQTT protocol. Two scenarios were made for this simulation. The first is to use a temperature interval equal to 1 s for the 5 devices. The second is to vary the sending intervals for each device (10 min, 5 min, 1 h, 30 min, 10 s).

**Fig. 5.**
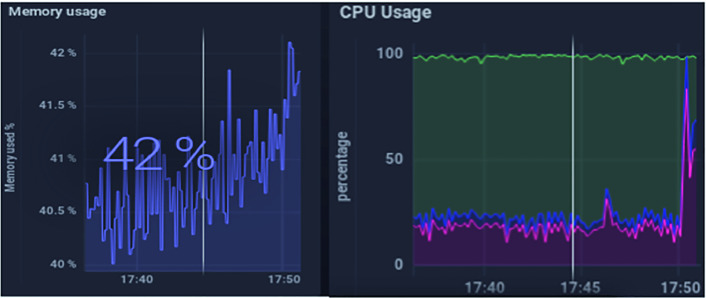
Evaluation of the fixed scenario

**Fig. 6.**
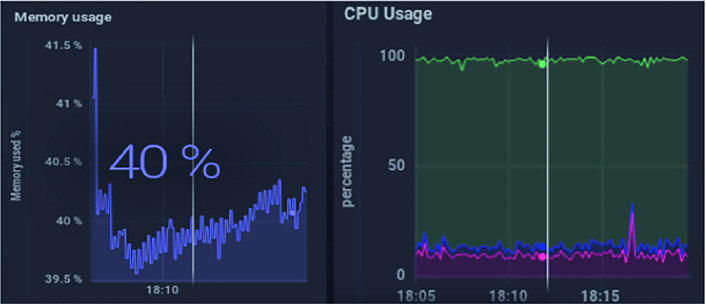
Evaluation of the self-configuration scenario

The evaluation results reflected in Fig. 5 and Fig. 6 are obtained using chronograf software which is an administrative tool for InfluxData deployments [16]. It shows that the use of a single data processing strategy in a fixed and non-custom way can result in an unnecessary use of gateway processor and memory which obviously affects performance and the time reaction specially in emergency cases.

## Conclusion and Future Work

In this paper, we proposed a self-adaptative early warning scores system that respect a risk evaluation approach named NEWS2. It provides a manual and self-adaptative configuration of the vital signs monitoring process depending on the patient health status variation and the medical staff decisions. We aim in our future work to use ontologies for more interpretation of collected data.
